# Can Aspirin Minimize Stroke Risk and New Lesion Formation in Multiple Sclerosis?

**DOI:** 10.3389/fneur.2018.00613

**Published:** 2018-08-08

**Authors:** Jagannadha Avasarala, Naveen Parti

**Affiliations:** ^1^Division of Neurology, Department of Internal Medicine, University of South Carolina School of Medicine, Greeville, SC, United States; ^2^Department of Radiology, University of South Carolina School of Medicine, Greenville, SC, United States

**Keywords:** aspirin, multiple sclerosis, stroke, perivenular lesion, relapse, MRI data

## Abstract

Even with increasing data implicating the venous side of the vascular tree of the brain in MS, no diagnostic or treatment protocol has addressed the risk of acute stroke in MS and no systematic study has documented the incidence or prevalence of acute strokein MS patients. Approximately 795,000 strokes occur in the U.S. each year—every 40 s, someone has a stroke and every 4 min, a person dies from a stroke. However, no large, prospective, multi-center study has investigated acute stroke incidence in MS patients either in the U.S. or internationally, leaving a gap in our understanding of the association between stroke and MS. Additionally, data on acute stroke in MS as determined by age, gender or ethnicity are unknown. To compound this further, the diagnosis and definition of acute stroke in MS remains poorly understood. A survey of published literature shows a few anecdotal reports of acute stroke occurring among MS patients, but most studies do not address the fundamental association between acute stroke and MS. Symptoms of acute stroke and MS can overlap and the lack of clear clinical/radiological criteria that alert the patient or clinician to the development of acute stroke in an MS patient compound the dilemma, even leading to the administration of IV alteplase in cases that are later diagnosed as either MS or having an “MS flare.” Clinical trials that use aspirin in multiple sclerosis are urgently needed.

Multiple sclerosis (MS) is a chronic inflammatory demyelinating disease of the central nervous system (CNS) and the commonest demyelinating disease of the CNS. Typically, MS lesions in brain are *perivenular* and a small vein occupies the center of the MS plaque (1). However, it is remarkable that the venous aspect of the disease remains the least explored entity from a clinical, pharmaceutical or pathological angle(s). Vascular aspects of MS (2–5) include impaired venous drainage, abnormalities of endothelial cells and arterial cerebral hypoperfusion. The walls of the veins in MS plaques are reported to be denser, heavily cellular and show perivenular fibrosis in the lesion center (6). Supporting the theory of venous vessel involvement in MS can also be gleaned from the use of 7 T MRI brain imaging that demonstrates the presence of a central vessel in 87% of visible white matter lesions (7). Deep medullary vein involvement in MS remains a cornerstone in the pathogenesis of focal MS lesions that is largely unexplored. However, research into the venous vessel wall involvement in MS is conspicuous by its absence despite the fact that patients with MS are at increased risk for cardiovascular disease or stroke. One of the many unanswered questions in MS research is the accumulation of disability which accrues with increasing age and vascular risk factors. Given the above, the lack of research into the vascular aspects of MS is startling.

Approximately 795,000 strokes occur in the U.S. each year—every 40 s, someone has a stroke and every 4 min, a person dies from a stroke. However, no large, prospective, multi-center study has investigated acute stroke incidence in MS patients either in the U.S. or internationally, leaving a gap in our understanding of the association between stroke and MS. Even with increasing data implicating the venous side of the vascular tree of the brain in MS, no diagnostic or treatment protocol has addressed the risk of acute stroke in MS and no systematic study has documented the incidence or prevalence of acute stroke in MS patients. Additionally, data on acute stroke in MS as determined by age, gender or ethnicity are unknown. To compound this further, the diagnosis and definition of acute stroke in MS remains poorly understood. A survey of published literature shows a few anecdotal reports of acute stroke occurring among MS patients, but most studies do not address the fundamental association between acute stroke and MS. Symptoms of acute stroke and MS can overlap and the lack of clear clinical/radiological criteria that alert the patient or clinician to the development of acute stroke in an MS patient compound the dilemma, even leading to the administration of IV alteplase in cases that are later diagnosed as either MS or having an “MS flare.”

Since epidemiological data suggest that patients with MS are probably at increased risk of developing ischemic stroke, better diagnostic paradigms for stroke in MS are urgently needed as risk of stroke increases with age.

Variations in study design and lack of standardization inherent in the differences in age/gender of populations being studied, and lack of datasets that track acute strokes in MS from large, multi-center MS centers hamper the true estimation of incidence/prevalence of acute stroke in MS across the disease spectrum. In a review of the incidence and prevalence of cerebrovascular disease in MS (8), the authors noted a summation of Scandinavian studies that revealed that the incidence for any type of stroke was 2.73%. In other studies (9, 10) prevalence varied from 0.4 to 7.0%. Some others reported a varying prevalence rates of stroke, ranging from 0.4 to 6.2%, based on population-based studies (10, 11). Although these numbers can be misleading and provide a limited view given patient heterogeneity, study quality, methodology and other limitations, stroke co-morbidity in MS patients is a true finding that impacts the overall health of MS patients.

The following are the challenges/opportunities to be considered for addressing stroke in MS patients,

1) Diagnosis of stroke remains clinical. Acute stroke symptoms are supposedly “sudden onset,” but not all symptoms meet that requirement—posterior circulation strokes, for example, can present with vague symptoms of dizziness, speech problems or weakness of an extremity in a subacute onset pattern; strokes that occur during sleep can often be misdiagnosed or missed, depending on presentation. Subtle symptoms such as nausea, numbness or headaches may escape scrutiny as not “typical for acute stroke.” Hence, following clinical symptomatology and evaluation alone particularly in MS patients may not raise the suspicion for acute stroke and most sudden-onset worsening in MS patients is typically dubbed as “MS flare.” Stroke and MS experts ought to define guidelines of how to identify a secondary disease such as acute stroke in MS patients.
2) Perfusion MRI scans may help but its utility even in the diagnosis of acute stroke lesions is uncertain, level U (12). Unfortunately, one of the most commonly used modalities of acute stroke lesion detection using MRI imaging, diffusion-weighted imaging (DWI), can also miss a significant percentage of posterior circulation strokes particularly if MRI is performed early, < 24 h (13). Moreover, DWI images in MS or stroke can have the same radiological features—high signal on DWI and low signal on an ADC map, making it difficult to establish a diagnosis. In some instances, intra-cytotoxic edema of acute plaques in MS can aid in identification of an acute lesion in MS but these changes are not universal. In summary, both clinical and radiological techniques have poor or limited sensitivity/specificity making detection of acute stroke in MS, challenging.
3) Prospective, double-blind, and placebo-controlled studies must be initiated to ascertain if MS patients benefit from routine use of anti-platelet drugs to prevent stroke. No single study has been published using aspirin (ASA) or any anti-platelet agent either alone or in combination with an approved disease-modifying agent for MS to evaluate if stroke risk or new lesion formation of MS can be favorably altered.
4) It should be noted that Zamboni's hypothesis (14) for treatment of chronic cerebrospinal venous insufficiency (CCSVI) via venous stenting was a total failure—the National MS Society (nmss.org) poured 2.4 million dollars into 7 new research projects jointly supported by the MS Society of Canada after an expedited international review. One of the hypotheses of CCVSI suggests that it could stretch venous walls, separate endothelial tight junctions causing extravasation of erythrocytes and iron deposition, a pro-inflammatory molecule, in the CNS parenchyma along venous drainage routes. Lower cerebral venous flow and reduced cerebral perfusion as well as endothelial dysfunction secondary to inflammation are thought to play a role in lesion development in MS. The role of fibrin in inflammation such as MS has been extensively studied (15), including links between BBB disruption and inflammatory demyelination, but specific therapeutic agents that downregulate vascular inflammation in MS have not been exploited.

Therefore, ASA use in MS patients could downregulate vascular inflammation via inhibition of platelet COX-1 and decrease in TXA_2_ synthesis, decreasing new lesion development around perivenular spaces. While ASA use reduces vascular risk of stroke, it could potentially also uncover novel therapeutic options in MS (Figure 1); it is a non-steroidal anti-inflammatory drug that is an irreversible inhibitor of cyclooxygenase (COX)-1 and COX-2. It upregulates the endogenous production of lipoxins which dampen inflammatory responsea and reduce levels of C-reactive protein, tumor necrosis factor-α and interleukin-6 (16). Therefore, ASA use may provide proof-of-principle for reducing inflammation and could address two diseases, stroke and MS, simultaneously. In the absence of any current algorithm to diagnose acute stroke in MS, the risk of use of low-dose ASA, particularly if no contraindications exist is a reasonable option. At a minimum, medical management to suppress venous vessel wall inflammation in MS is worth pursuing via well-designed clinical trials that combine ASA and a disease-modifying drug vs. disease-modifying drug alone.

**Figure 1:**
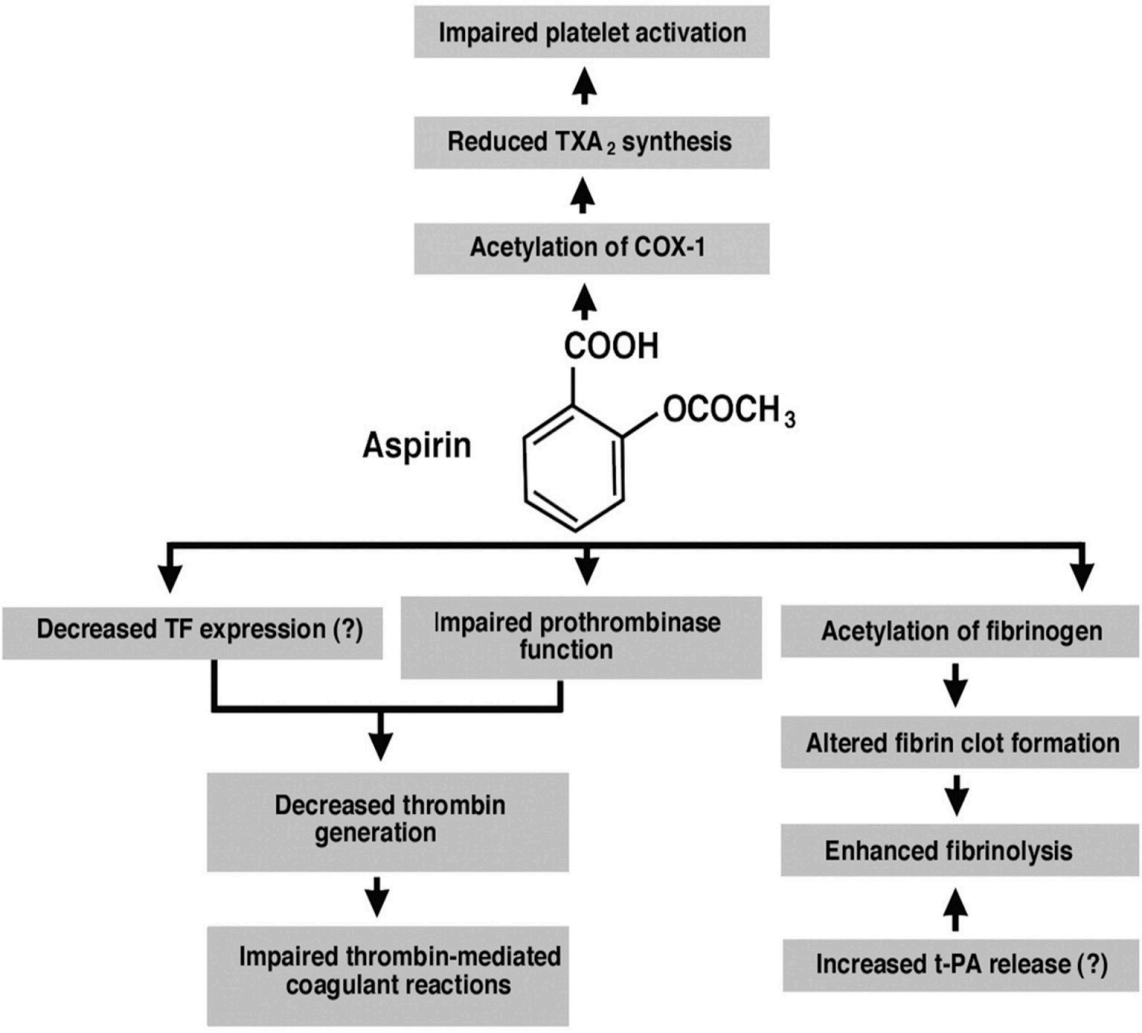
Showing ASA mode of action.

## Access to data

Both the authors listed had access to all the data in this manuscript.

## Author contributions

JA: writing the article, concept, idea, and interpretation. NP: MRI data evaluation and analysis.

### Conflict of interest statement

The authors declare that the research was conducted in the absence of any commercial or financial relationships that could be construed as a potential conflict of interest.
